# Supplementary material for: Development of a Tailored, Complex Intervention for Clinical Reflection and Communication about Suspected Urinary Tract Infections in Nursing Home Residents
Source: Antibiotics (Basel). 2020 Jun 25;9(6):360. doi: 10.3390/antibiotics9060360 (PMC7345997; doi:10.3390/antibiotics9060360)
Supplement: Supplementary file 1 [file antibiotics-09-00360-s001.zip › supplementary materials/Text B1.pdf]

Case, Part 1: The Reflection Tool

Imagine you are caring for this resident:

Anna Andersen (Social Security Number: 123456-7890) is an 89-year-old woman with dementia. When you help her into her clothes, she seems more confused than usual and at first believes that you are her granddaughter. Her urine smells and is cloudy and continues to be so when you help her to the toilet later that morning. Anna is self-reliant - except for dressing and toilet visits – and today, as any other day, she walks around the nursing home with a cane. She does not use a urinary catheter.

You do a urinary dipstick test that shows nitrite and leucocytes (bacteria). Her temperature is 37 °C, blood pressure 105/60 and pulse 85.

Case, Part 2: The Communication Tool

The next day she is even more confused and does not want to get out of bed. Otherwise, your observations and vital signs are unchanged. You chose to contact the physician.
