# Supplementary material for: Development of a Tailored, Complex Intervention for Clinical Reflection and Communication about Suspected Urinary Tract Infections in Nursing Home Residents
Source: Antibiotics (Basel). 2020 Jun 25;9(6):360. doi: 10.3390/antibiotics9060360 (PMC7345997; doi:10.3390/antibiotics9060360)
Supplement: Supplementary file 1 [file antibiotics-09-00360-s001.zip › supplementary materials/Text S1_complete teaching material.pdf]

SUSPECTED URINARY TRACT INFECTION

Communication, Diagnosis and Treatment

MD, PhD student Sif Helene Arnold

Hello and thank you for having me. My name is Sif Arnold and I am an MD and Ph.D. student.

We are here today because you have agreed to participate in a project about communication, diagnosis and treatment of suspected urinary tract infection (UTI). There are 22 participating nursing homes and if the project is a success the way to approach suspected UTI you are learning today could be used in all nursing homes in Denmark. So thanks again for participating.

I would like to start by saying that you have a tough job out there. And unfortunately I don´t have a guaranteed solution, but I think that what is needed to eliminate at least some of the UTIs is to engage your professional capabilities.

In the project period, every time you suspect a UTI, you have to use a dialogue tool, that supports your professional skills and structures the dialogue with the physician.

With regards to this session, we are many different professionals gathered here today, so some things I say may surprise you, and others might not. I hope you will participate actively in the discussion regardless.

PROGRAM

Background of project and dialogue tool

The dialogue tool

Case 1: A UTI?
I use the dialogue tool

Case 2: A UTI?
You use the dialogue tool

Practicalities

This is the program for today.

>Read through program<

And please remember that all questions are welcome.

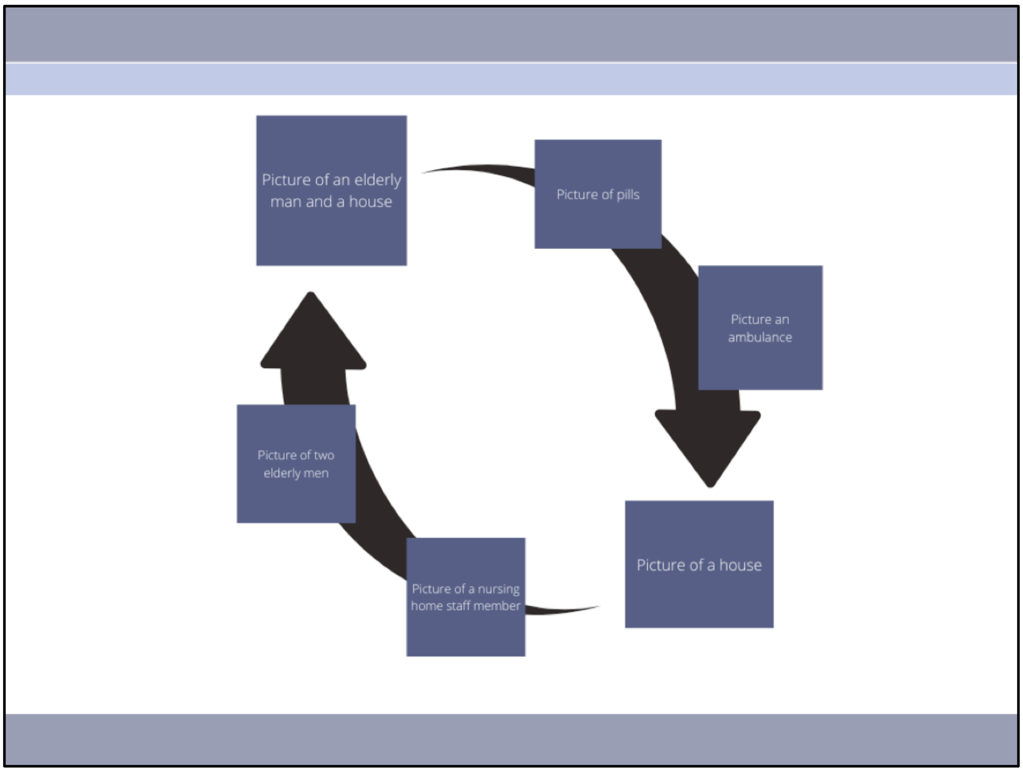

I would like to start by talking about, why it is important to talk about suspected urinary tract infections. Basically, it is because we treat a lot of them. Some of the treatments could have been avoided, and that has consequences. I would like to give you an example of that from a nursing home that I have visited:

This is Adam. He has dementia and lives in a nursing home. During the heat wave last summer, Adam starts to seem more confused than usual and his urine starts to smell funny. The urine dipstick test is positive. The nursing home calls the physician, who starts him on antibiotics just in case and sends the urine sample to the hospital for testing. The heat wave ends and Adam starts to feel better the next couple of days, but when the test results comes back it turns out that the bacteria in Adams urine is resistant to the antibiotics he has been given. The physician prescribes another broader type of antibiotics for Adam. But Adam has a hard time tolerating this new medication. He gets nausea and diarrhoea and has a hard time eating and drinking. Because he is a small guy, it is not long before he is so dehydrated that he needs to be admitted to the hospital. At the hospital they test Adam, and discovers that he harbours multi-resistant bacteria. He is eventually discharged, but now has to be shielded in his home. But it is not long till the resistant bacteria spread to two of the other residents at the nursing home, who also must be isolated.

There are many points to be drawn from this example, but I would like you to notice this:

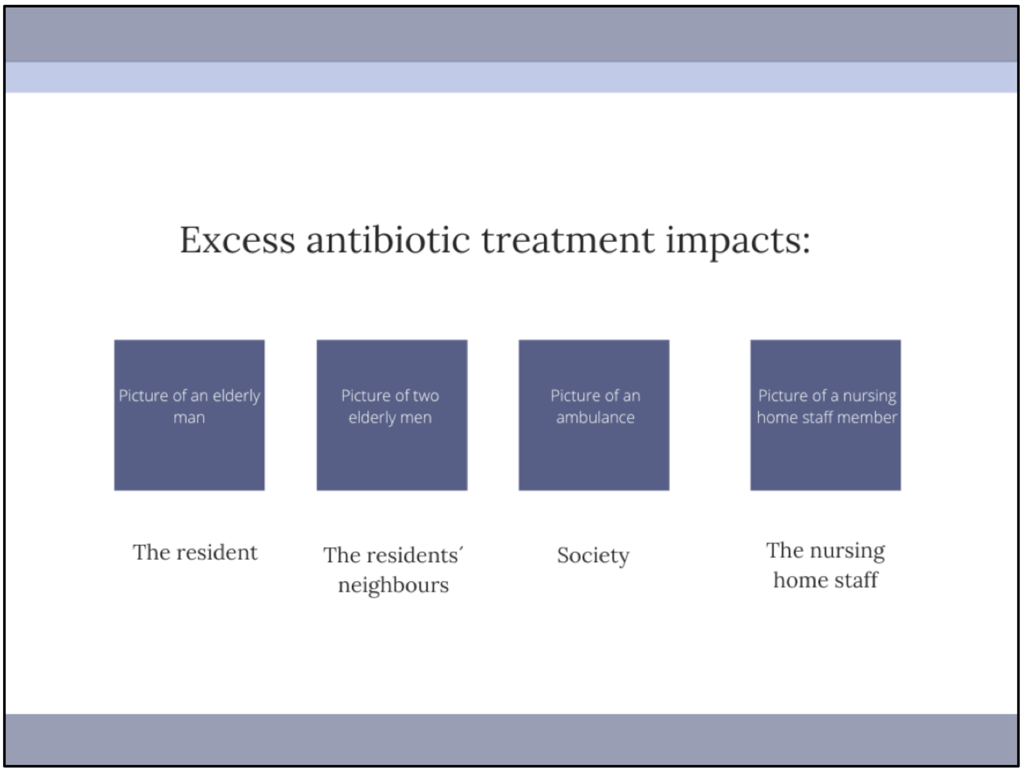

Antibiotic treatment can have severe consequences; for the treated resident, who becomes really ill; for those closest to the resident, who can be infected: for society that has to pay for tests and isolation: and for you, the nursing home staff, who will experience a bigger workload.

Off course we have to use antibiotics for those who are really ill. But the problem is that those who have a ”true” UTI can be hard to spot. The reason it is difficult is that the residents can be hard to observe and the communication pathway from resident to physician is really complex at the nursing homes.

You all know it, but let me show you what it looks like in a drawing.

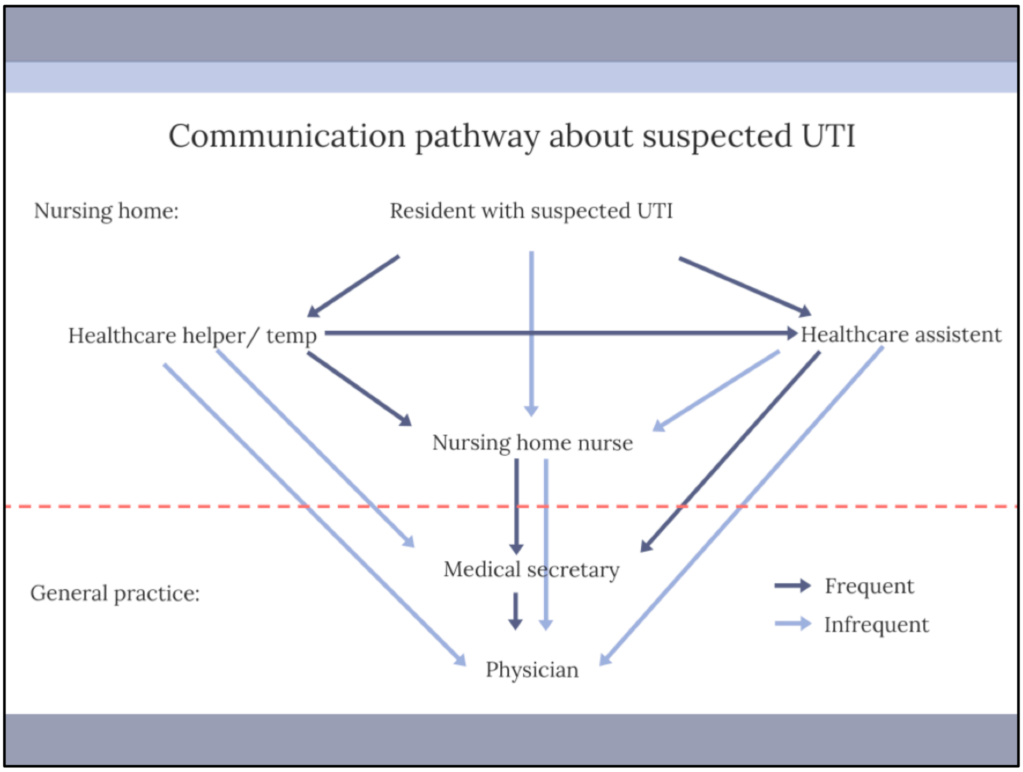

What you see here is the communication pathway from resident with suspected UTI to the physician. There are many different paths from resident to physician, but the dark blue arrows show the most common communication pathways and the light blue arrows show the rare communication pathways.

There are three important takeaways from this figure:

1. Firstly, the communication pathway has many links. Typically, a healthcare helper or assistant will suspect the UTI. A healthcare helper is typically not allowed to call the physician and the observations are passed on to an assistant or a nurse. When you suspect the UTI it is often late morning, so the one that contacts general practice will get a hold on the medical secretary, who then passes the information on to the physician. This reminds me very much of a whispering game I used to play when I was little. You probably know it. I start by whispering a sentence to you, you whisper the sentence to your neighbour, who whisper to his neighbour, until the sentence has passed through all of us. My point is that we all have to make an effort, if the sentence we end up with is the same sentence we started with. The same goes for the clinical information.
2. Secondly, you need to notice that the resident and the physician typically are at two separate physical addresses: The nursing home and the general practice. This means that it is your observations and assessments that determines the diagnosis.
3. Thirdly, this communication pathways only goes one way. If the physician lacks information about the resident, the communication pathway must be reversed. That is tedious for you, but also for the physician.

Therefore, I have developed a dialogue tool to support you in your observations and the communication with the physician.

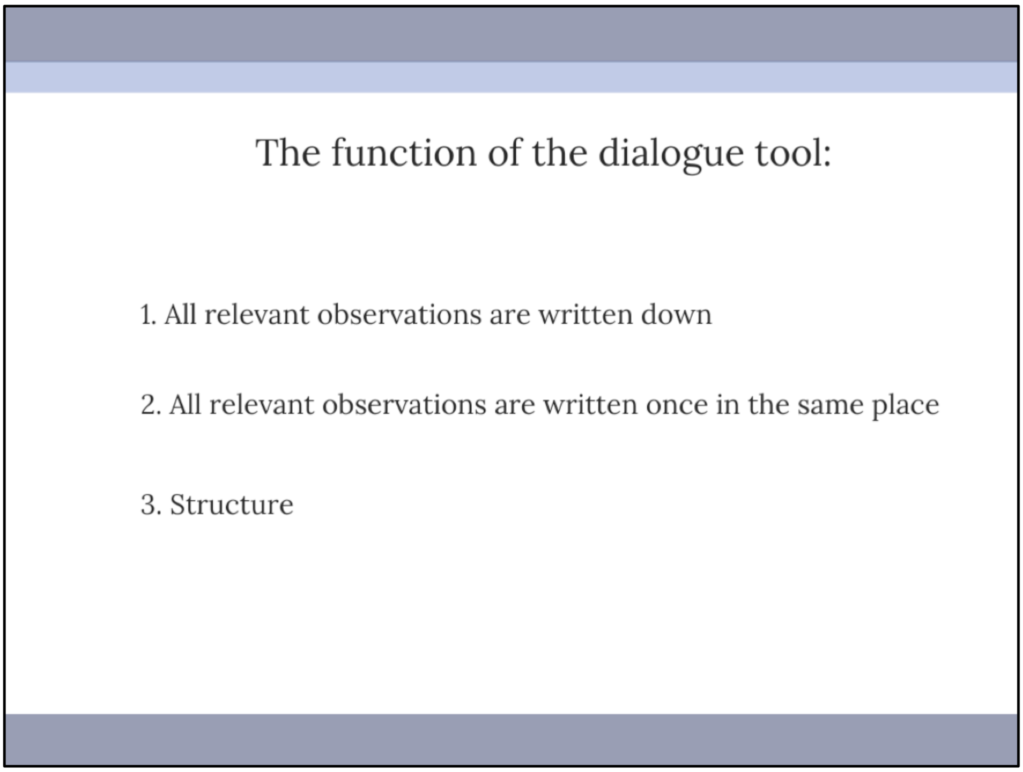

Therefore, the function of the dialogue tool is: to write down all relevant observations, to write down the relevant observations once in the same place, and to provide structure to the communication with the physician.

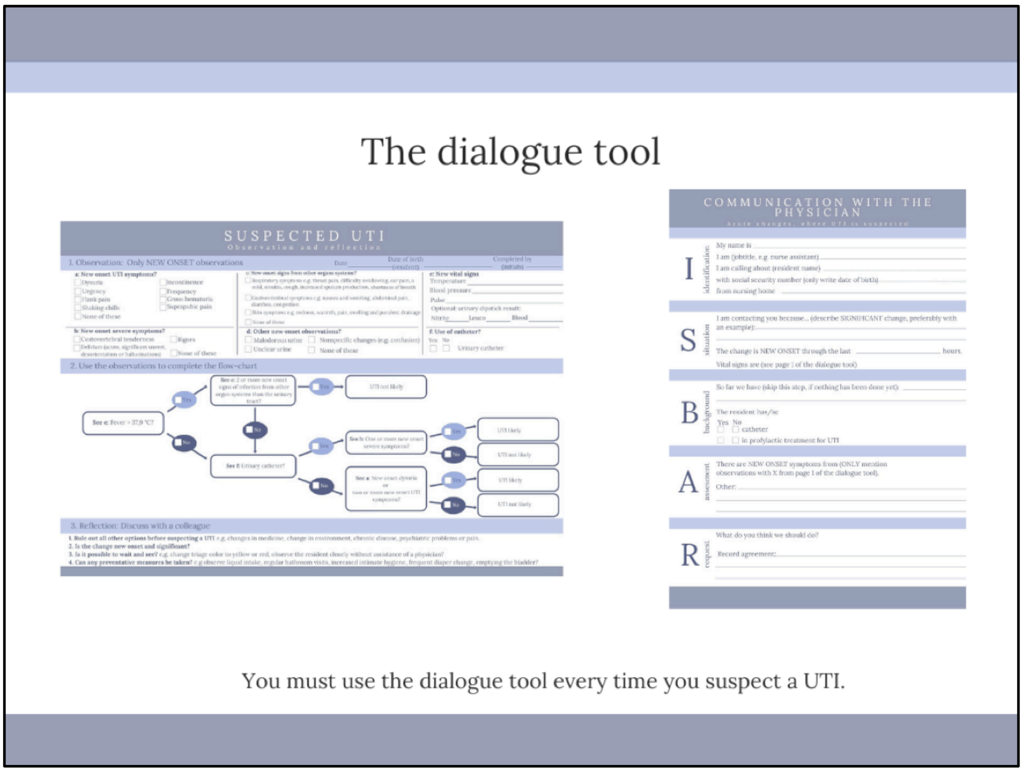

Now we will dive deeper into the details of the dialogue tool.

It looks like this and you also have one in front of you.

The dialogue tool is developed through a collaboration with nursing home staff from several different nursing homes, general practice, residents and their relatives.

On the first page, there is an observation and reflection tool. On the second page, there is a communication tool for use when you are communicating with the physician. It is structured as an ISBAR that stands for Identification, Situation, Background, Assessment and Recommendation.

The dialogue tool is build around three key points that we are going to discuss in a short while. But before we do that I would like you to answer the following question:

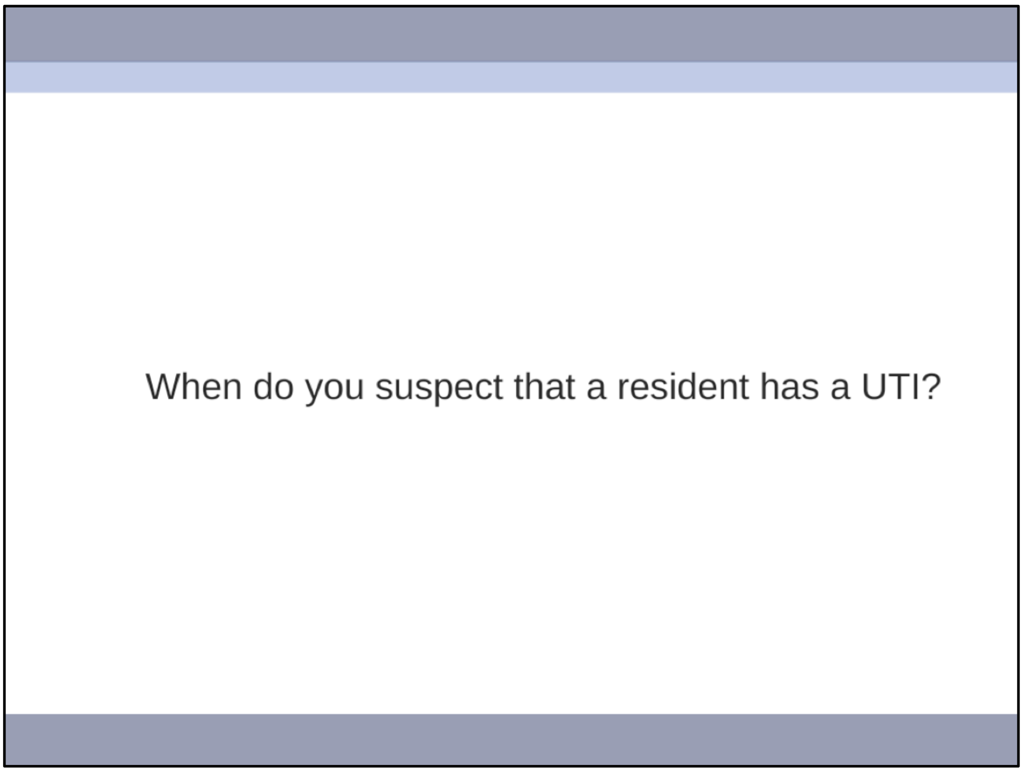

When do you suspect that a resident has a UTI?

>Leave time for answering the question<

From your answers I gather that you can divide the symptoms you are describing into four groups:

1. Specific symptoms like “pain when urinating” and going to the toilet more often.
2. Nonspecific symptoms like confusion and falls.
3. Signs of bacteria in the urine like a positive dipstick, smelly and unclear urine
4. Severe symptoms like severe back pain or signs of delirium.

But not all of them actually constitutes a UTI.

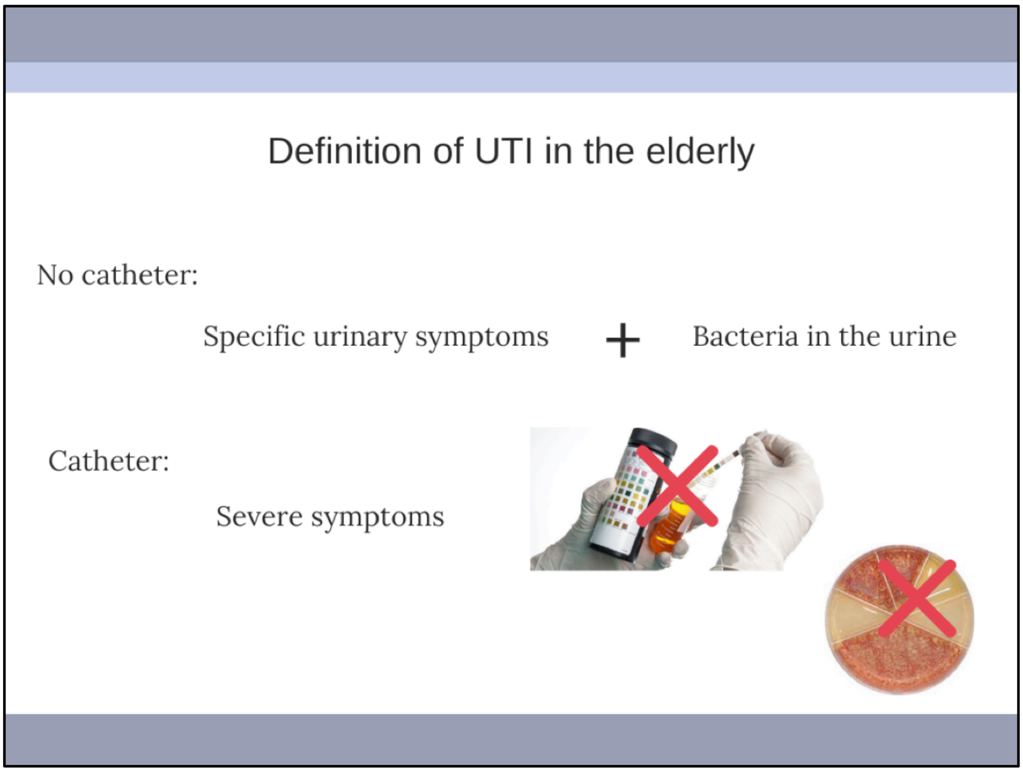

First of all, there is a difference between residents with and without a urinary catheter.

If you do not have a catheter there needs to be both specific urinary symptoms like frequent urination AND signs of bacteria in the urine like a positive dipstick, smelly or unclear urine before you can say that the resident has a UTI.

If you have a catheter, then there needs to be severe symptoms like severe back pain, shaking chills or delirium, that shows itself as marked unrest or hallucinations before you can say that the resident has a UTI.

The reason that it is important to stick to these definitions is that the older we get, the more common it is to have bacteria in the urine habitually. In fact, up to 50% of those residents without a catheter and 100% of those with a catheter has bacteria in the urine. That means that if we did a dipstick test on all your healthy residents half would have a positive dipstick and of the residents with a catheter all of their dipsticks would be positive, but none of them would have a UTI. There are many studies that document that bacteria in the urine is not dangerous and should not be treated. Therefore, you can´t say that if you have a positive dipstick, you have a UTI. The same goes for a positive urine culture. There must be some sort of symptom related to the urinary tract before you can say it is a UTI.

Therefore, I have been a little bit provocative and crossed out both the urinary dipstick and the urinary culture, so that you remember that a positive urine sample is not the same as a UTI.

But where do the nonspecific symptoms then factor into this definition? Before we talk more about this, I would like you to answer the following question:

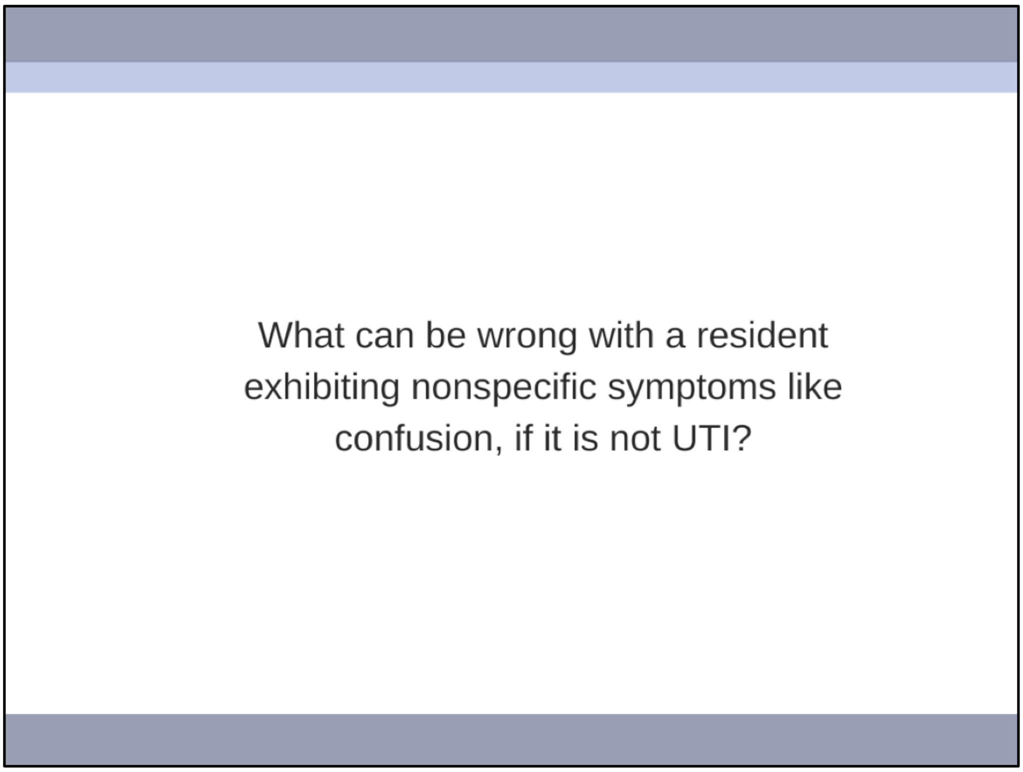

What can be wrong with a resident exhibiting nonspecific symptoms like confusion, if it is not UTI?

>Leave time for answering the question<

Hopefully, you agree with me that a hoist of other things than UTI can be wrong with this patient.

We don´t know exactly, how many elderly with nonspecific symptoms have a UTI, but it probably is a very small percentage. One study showed that if you had 100 nursing home residents that exhibited nonspecific symptoms 25 of them would need medical attention and the others would get better by themselves. We don´t know how many of these had a UTI, but it was an even smaller part than the 25.

The residents with nonspecific symptoms often are very hard to asses and therefore, we may be a little quick with prescribing antibiotics.

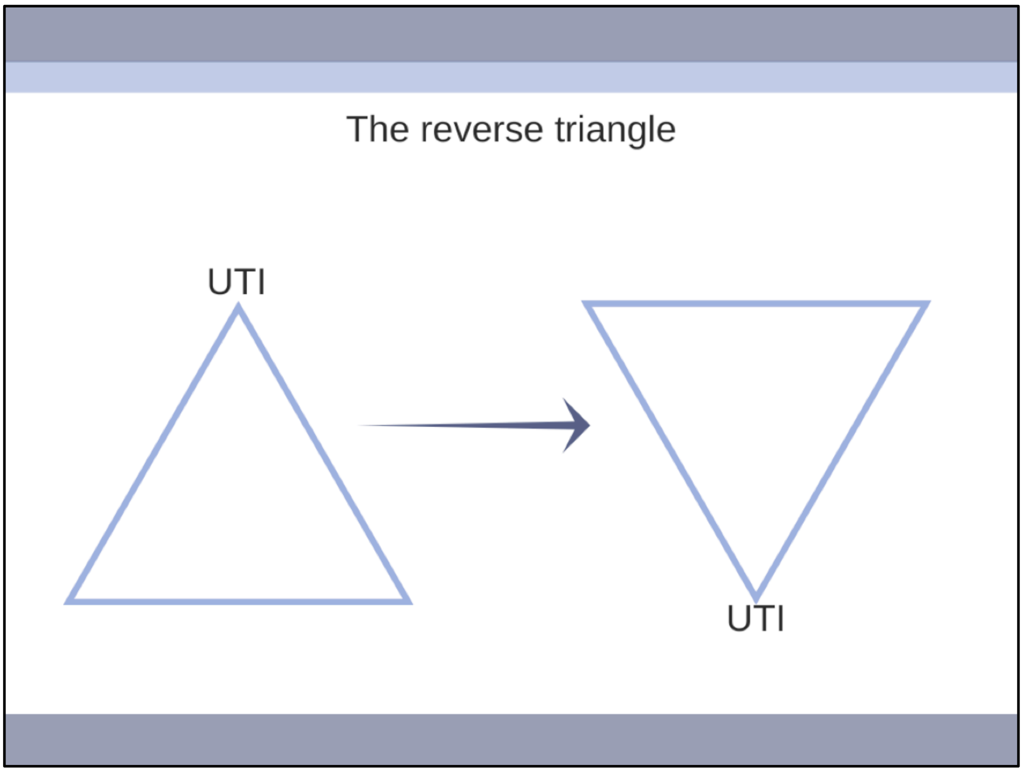

Today we have a tendency to think UTI first. It is only when the treatment does not work that we consider other possibilities. It is important to think the other way around that is, when symptoms arise, it is important that you consciously use your professional knowledge and think about if it could be something else than a UTI, because UTI is a diagnosis of exclusion. That means that you first have to exclude other possible diagnosis and only then consider UTI. Today we have a tendency to do the reverse and so we end up treating many UTIs, when actually something else is wrong. I call this way of thinking ”the reverse triangle”.

The reverse triangle is the first step in describing and considering symptoms, when you suspect a UTI. It looks like this:

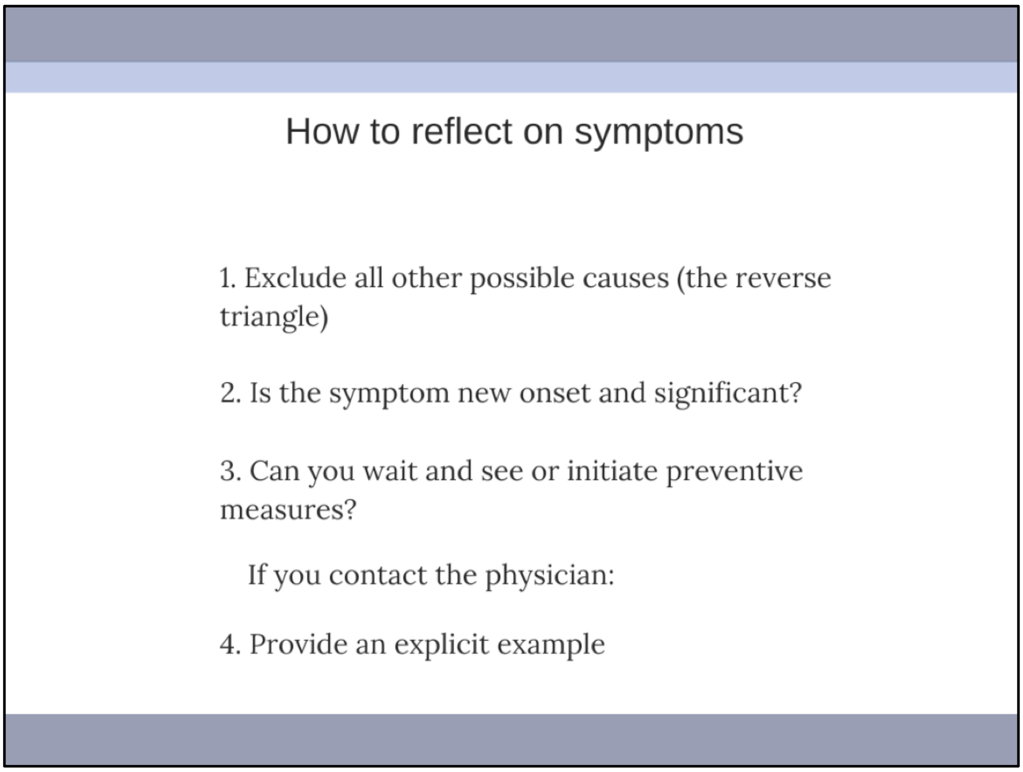

First of exclude all other possible causes.

Then you need to decide if the symptoms are new onset and significant. UTIs are acute that means they have a sudden onset and is so bothersome that you are not in doubt about whether or not to react. If this is not the case, then there is a large risk that it could be something else than UTI.

Then you have to decide if you can wait and see or initiate some preventive measures. If a resident for example is dehydrated and therefore not himself, you could wait and see while providing fluids to rebalance. In this case, time could be a valuable factor in the cure.

If you contact the physician, then you have to help him understand your concerns and provide an explicit example of how the resident acts, especially if they exhibit nonspecific symptoms. This will make it easier for the physician to assess the seriousness of the situation.

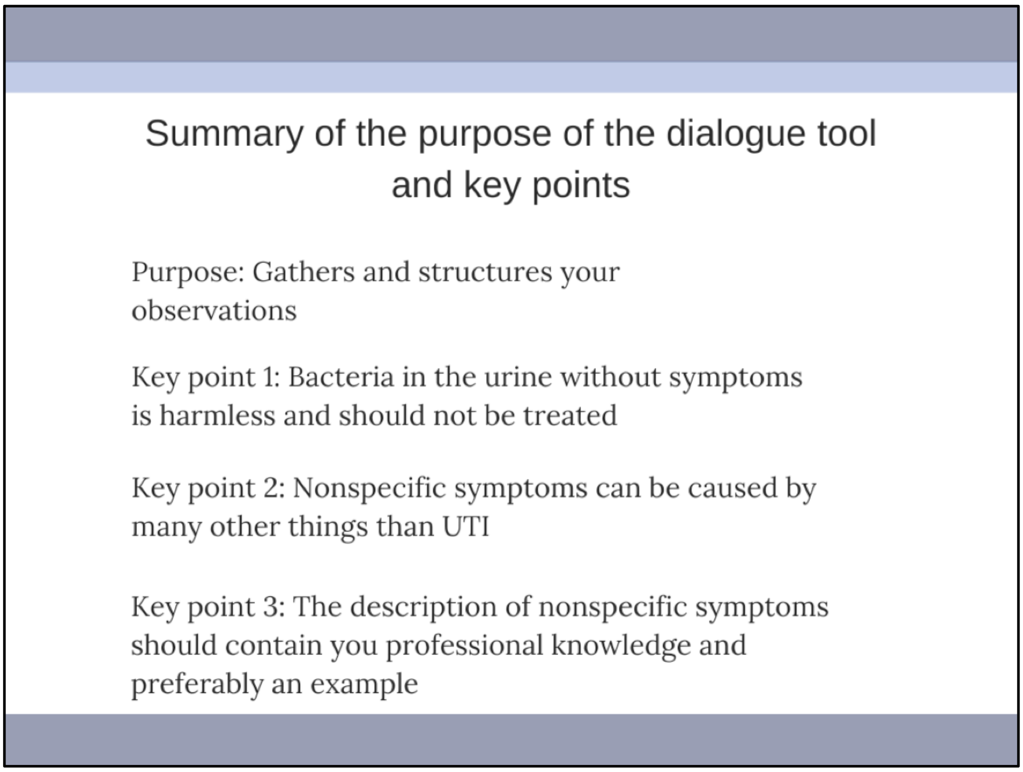

So the purpose of the dialogue tool is to gather and structure your observations as well as remind you of the three main points that we have discussed so far. These are:

>Read from slide<

Next I am going to use a case to familiarize you with the dialogue tool.

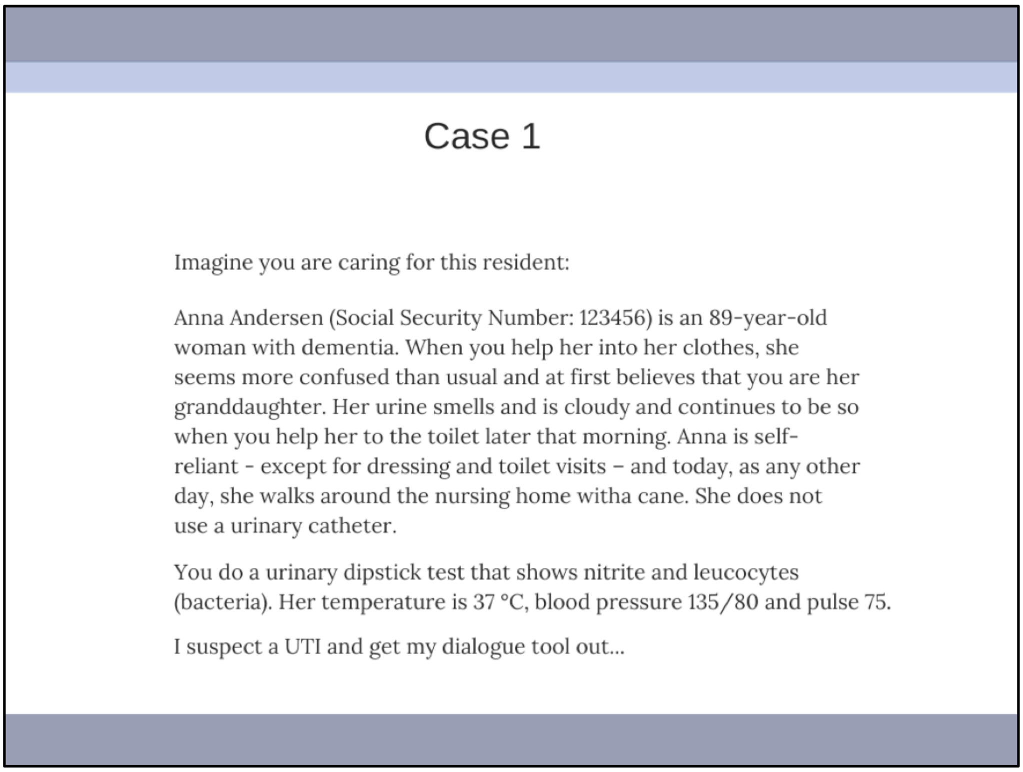

Case 1 sounds like this:

>Read Case aloud<

Next, I am using the dialogue tool using the case. The purpose is so you can see how I do it step by step and hear my considerations. It is important to note that I have another background than you, so you might have evaluated the case differently and done something different from me. I would like you to consider this while I talk and then we can discuss it afterwards.

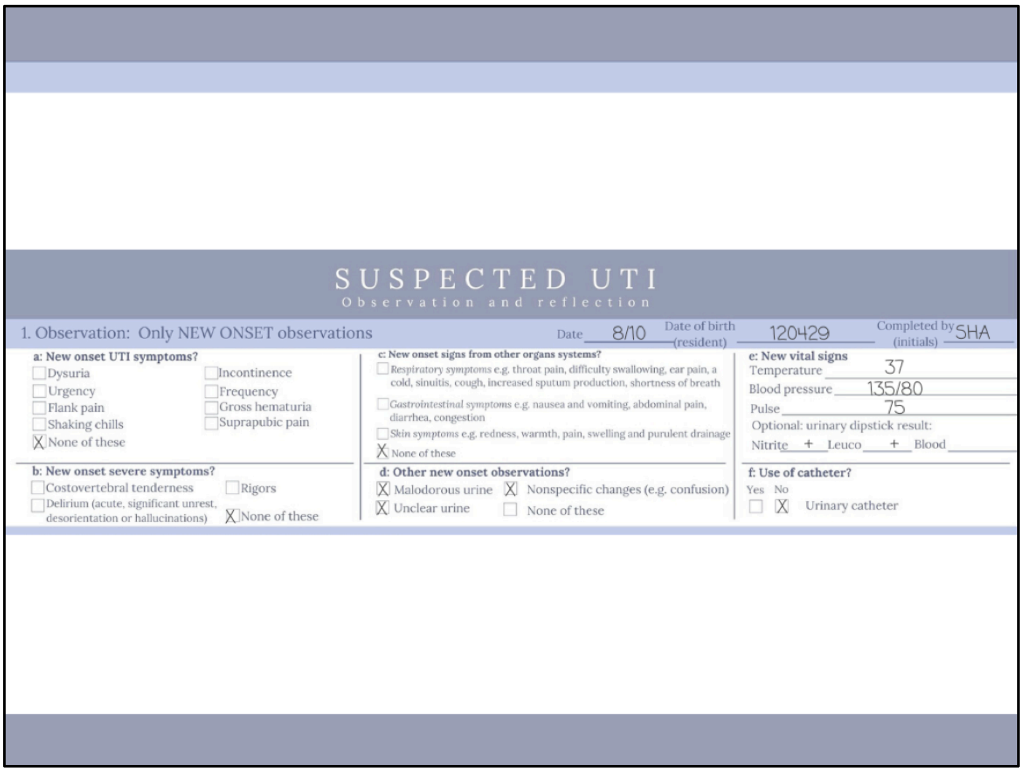
SUSPECTED UTI
Observation and reflection

1. Observation: Only NEW ONSET observations — Date 8/10 — Date of birth (resident) 120429 — Completed by (initials) SHA

a: New onset UTI symptoms?
- [ ] Dysuria
- [ ] Urgency
- [ ] Flank pain
- [ ] Shaking chills
- [X] None of these
- [ ] Incontinence
- [ ] Frequency
- [ ] Gross hematuria
- [ ] Suprapubic pain

b: New onset severe symptoms?
- [ ] Costovertebral tenderness
- [ ] Delirium (acute, significant unrest, desorientation or hallucinations)
- [ ] Rigors
- [X] None of these

c: New onset signs from other organs systems?
- [ ] Respiratory symptoms e.g. throat pain, difficulty swallowing, ear pain, a cold, sinuitis, cough, increased sputum production, shortness of breath
- [ ] Gastrointestinal symptoms e.g. nausea and vomiting, abdominal pain, diarrhea, congestion
- [ ] Skin symptoms e.g. redness, warmth, pain, swelling and purulent drainage
- [X] None of these

d: Other new onset observations?
- [X] Malodorous urine
- [X] Unclear urine
- [X] Nonspecific changes (e.g. confusion)
- [ ] None of these

e: New vital signs
Temperature 37
Blood pressure 135/80
Pulse 75
Optional: urinary dipstick result:
Nitrite + Leuco + Blood

f: Use of catheter?
Yes [ ] No [X] Urinary catheter

I start with the observation and reflection tool. First I note the date, the residents date of birth and my initials.

In the first part I gather all my observations in one place.

I also have to remember that all symptoms must be new onset and then I have to find out if there are any new onset urinary symptoms. There are none of these.

So I must consider if there are any severe symptoms. There are no new onset back pain, Rigors or signs of delirium.

Then I think about new onset signs from other organ systems. There are no respiratory symptoms e.g. Cough, no gastrointestinal symptoms like vomiting and there are no skin symptoms like redness. So there are none of these.

Are there other new onset symptoms? Yes, in fact there are signs of bacteria in the urine, because she has smelly and unclear urine and there are new onset nonspecific changes because she is more confused than usual.

She has a normal temperature of 37 degrees, blood pressure of 135/80 and a pulse of 75 BPMs and a positive urine dipstick.

She does not use a catheter.

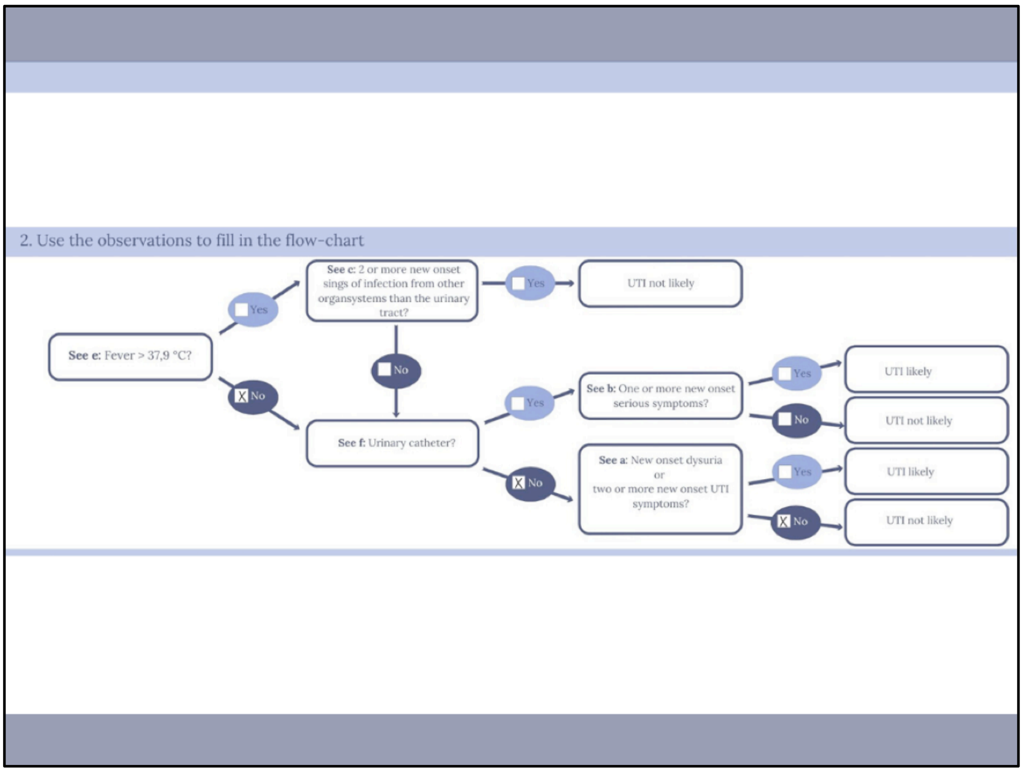

In the second part you have to use the observations to reach a conclusion in the flowchart.

Firstly, you have to look at your observations and see if Anna had a fever. No she did not because her temperature was 37 degrees.

Then you have to remember if she had a urinary catheter. No, she did not.

Then you consider if she had newly onset dysuria or two or more new onset urinary symptoms. No, she had none of these.

According to the flowchart UTI is not likely in Anna's case. And when you look back on the information that we gathered I can see that it is because Anna has some nonspecific symptoms and signs of bacteria in the urine that I get worried. But bacteria in the urine without urinary symptoms in itself is harmless and should not be treated. But the nonspecific symptoms should be considered more thoroughly, so we jump to the third part of the observation and reflection tool.

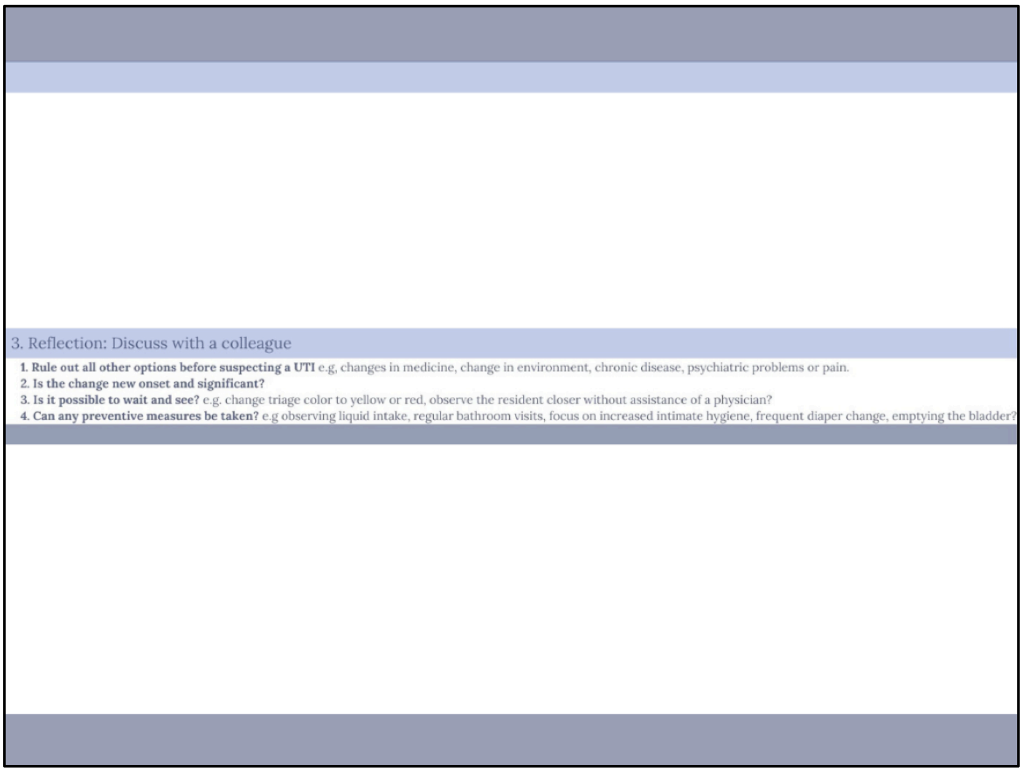

In the third part your professional abilities has to come into play. As often as possible discuss the four bullet points with a colleague.

First consider if there are other causes of Anna's confusion, than those we spoke of earlier like medical changes, an event that could have caused a change in mood, chronic disease, psychiatric problems or pain or other things.

Then consider if the change is new onset and significant.

How bad is it? Can you make new observations of Anna in a couple of hours or tomorrow to see if the symptoms pass on their own?

Can you initiate other preventive measures like observing liquid intake, regular bathroom visits, focus on increased intimate hygiene, frequent diaper change or something else before contacting the physician?

In this case I would say that Anna could be dehydrated. So the change is new onset but not significant, so maybe we could wait and see what happens with Anna while we help her get more fluids to rid her of her smelly urine.

What are you thoughts?

>Short discussion<

Case 1 -continued

You chose to observe Anna and try to increase her liquid intake.

The next day, she is more confused and does not want to get out of bed. She has consumed 1L of fluids since yesterday at noon.
Her temperature is 37,5, blood pressure 137/72 and pulse 80.
The urine is still smelly and unclear and the dipstick is positive for nitrite and leucocytes (bacteria).

You still suspect a UTI and take out your dialogue tool...

So I chose to observe Anna and try to increase her liquid intake. The case continues the next day and by then the situation has changes a bit.

>Read the case aloud<

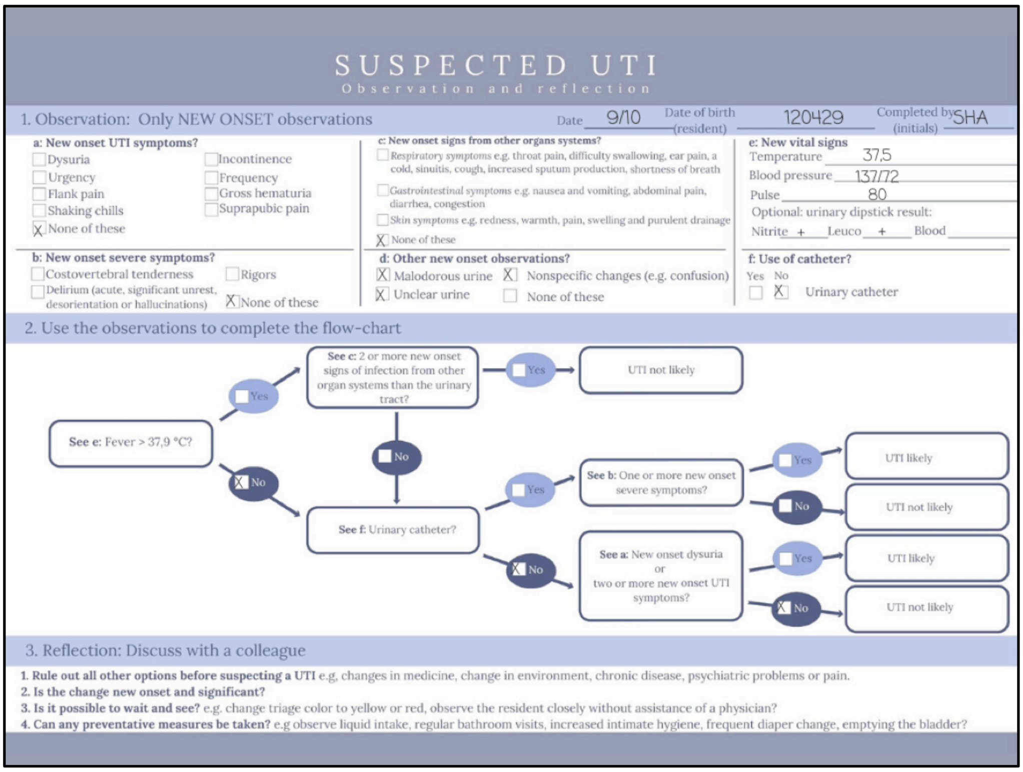

SUSPECTED UTI
Observation and reflection

1. Observation: Only NEW ONSET observations — Date 9/10 — Date of birth (resident) 120429 — Completed by (initials) SHA

a: New onset UTI symptoms?
- [ ] Dysuria
- [ ] Urgency
- [ ] Flank pain
- [ ] Shaking chills
- [X] None of these
- [ ] Incontinence
- [ ] Frequency
- [ ] Gross hematuria
- [ ] Suprapubic pain

c: New onset signs from other organs systems?
- [ ] *Respiratory symptoms* e.g. throat pain, difficulty swallowing, ear pain, a cold, sinuitis, cough, increased sputum production, shortness of breath
- [ ] *Gastrointestinal symptoms* e.g. nausea and vomiting, abdominal pain, diarrhea, congestion
- [ ] *Skin symptoms* e.g. redness, warmth, pain, swelling and purulent drainage
- [X] None of these

e: New vital signs
Temperature 37,5
Blood pressure 137/72
Pulse 80
Optional: urinary dipstick result:
Nitrite + Leuco + Blood

b: New onset severe symptoms?
- [ ] Costovertebral tenderness
- [ ] Delirium (acute, significant unrest, desorientation or hallucinations)
- [ ] Rigors
- [X] None of these

d: Other new onset observations?
- [X] Malodorous urine
- [X] Unclear urine
- [X] Nonspecific changes (e.g. confusion)
- [ ] None of these

f: Use of catheter?
Yes [ ] No [X] Urinary catheter

2. Use the observations to complete the flow-chart

3. Reflection: Discuss with a colleague

1. **Rule out all other options before suspecting a UTI** e.g, changes in medicine, change in environment, chronic disease, psychiatric problems or pain.
2. **Is the change new onset and significant?**
3. **Is it possible to wait and see?** e.g. change triage color to yellow or red, observe the resident closely without assistance of a physician?
4. **Can any preventative measures be taken?** e.g observe liquid intake, regular bathroom visits, increased intimate hygiene, frequent diaper change, emptying the bladder?

I tick the same boxes in the observation and reflection tool, because nothing but the nonspecific symptoms have changed since yesterday.

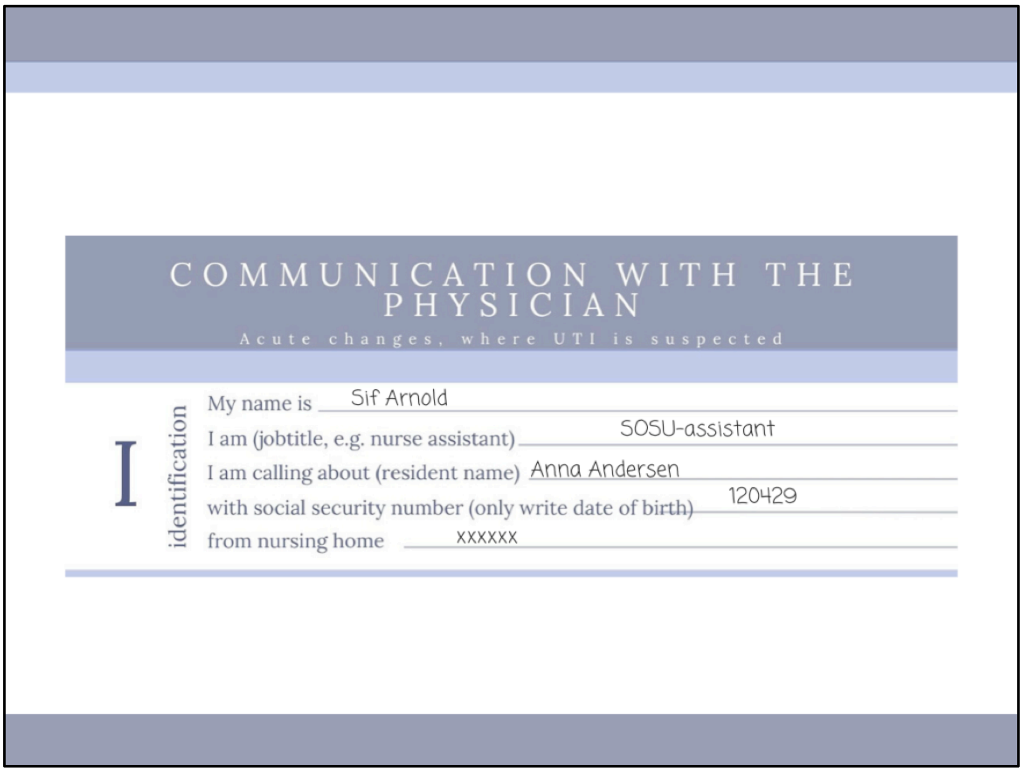

The communication tool is made for communicating over the phone, so when all information is written, you can just read aloud. But if you communicate with the physician by edifact (email, red.) you just copy what you wrote into it.

The first part of the communication tool is where I identify myself and the resident.

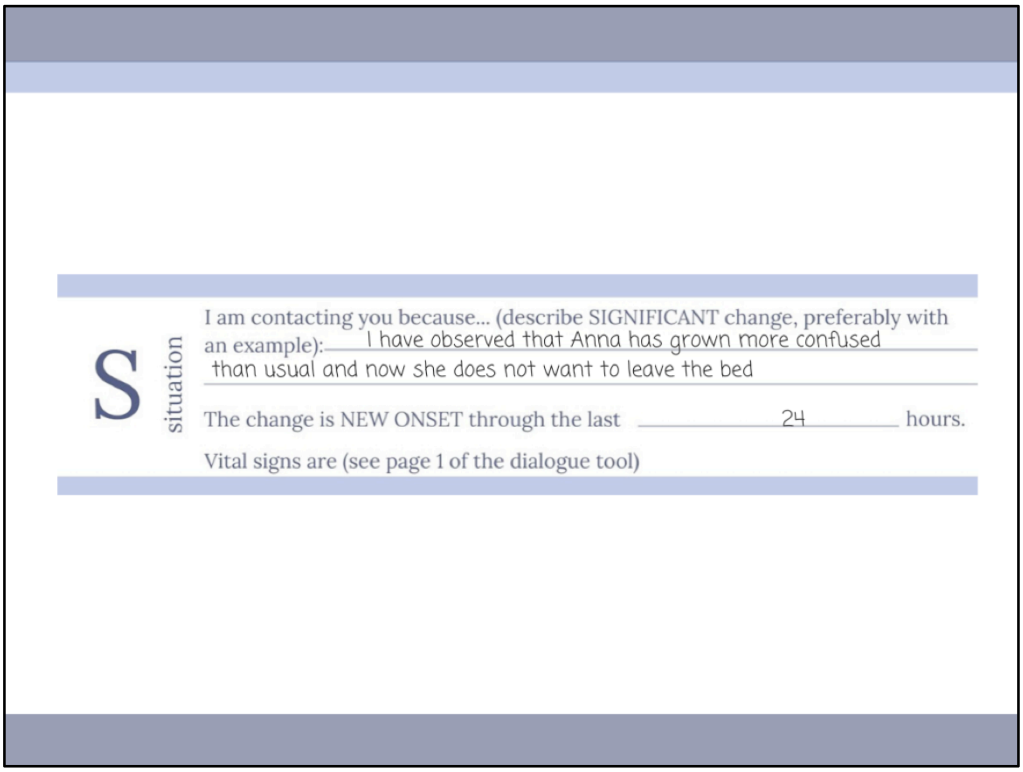

S situation

I am contacting you because... (describe SIGNIFICANT change, preferably with an example): I have observed that Anna has grown more confused than usual and now she does not want to leave the bed

The change is NEW ONSET through the last 24 hours.

Vital signs are (see page 1 of the dialogue tool)

Then I must describe the situation. I do this by giving an example of what the nonspecific changes are and how they affect her. So I would say: >Read aloud from form<.

The change has occurred within the last 24 hours and is therefore new onset.

I have measured the vital signs in the observation and reflection tool.

Then I move on to Background.

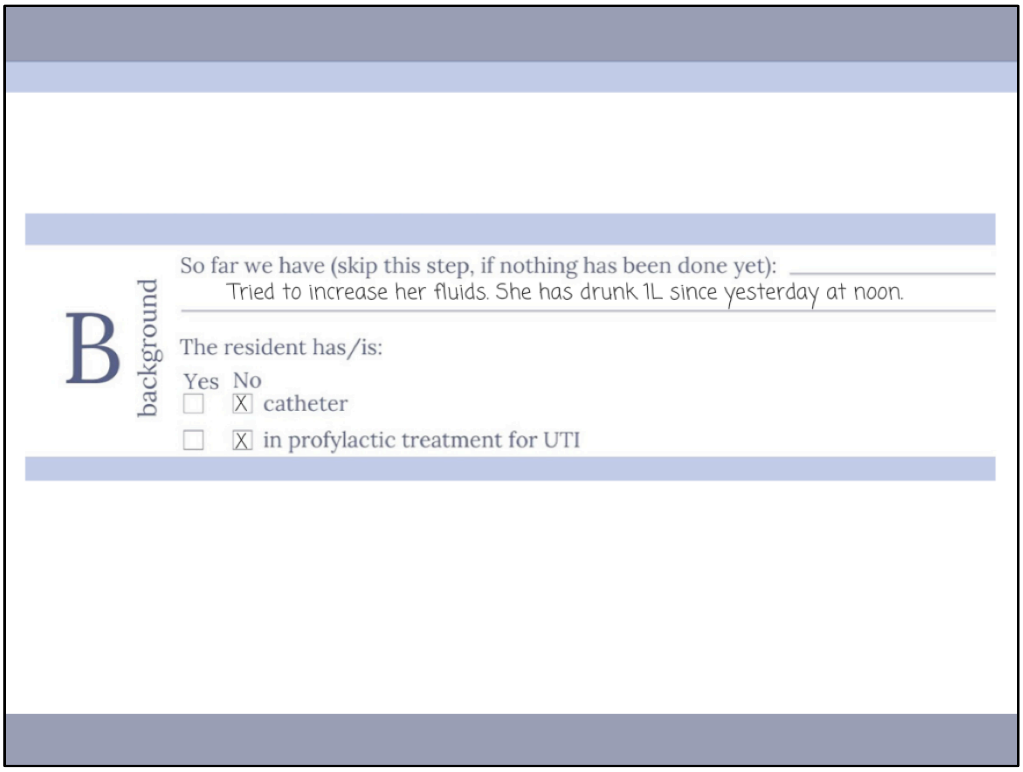

So far we have tried to increase her fluids and she has drunk 1L since yesterday at noon, She does not use a catheter and is not in prophylactic treatment for UTI.

Next is the assessment.

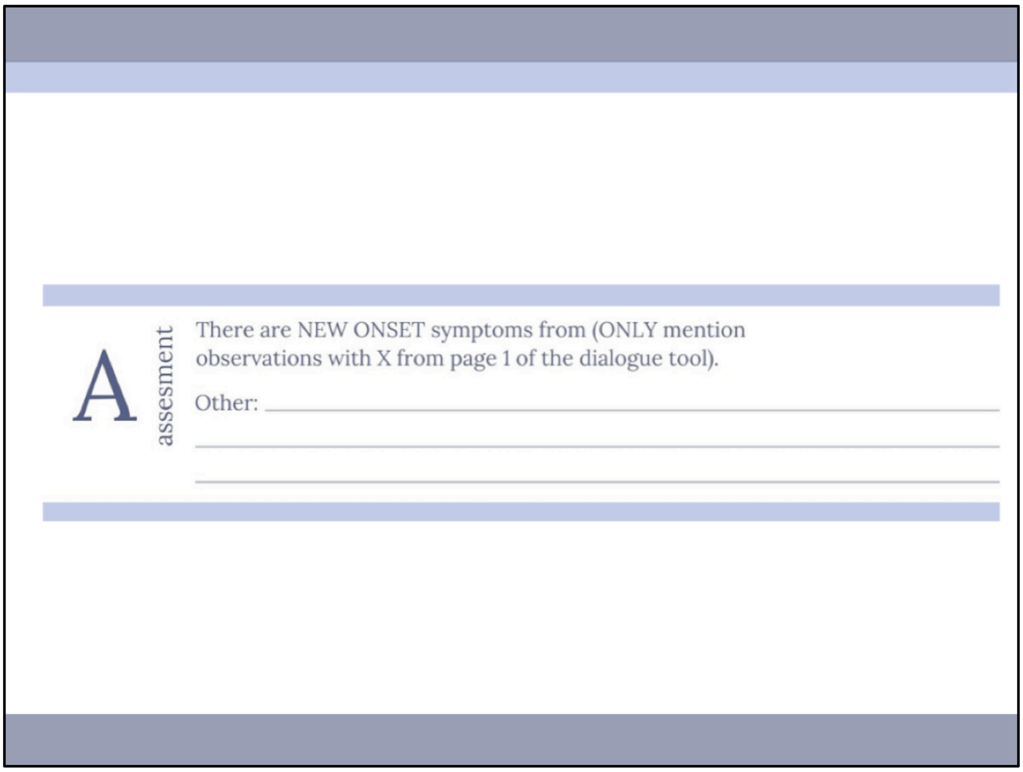

Here I need to check if there are new onset symptoms from the urinary tract or other places on page one of the dialogue tool. In this case we see signs of bacteria in the urine with a positive urinary dipstick and smelly and unclear urine.

She has also grown significantly more confused.

Lastly, we need a recommendation.

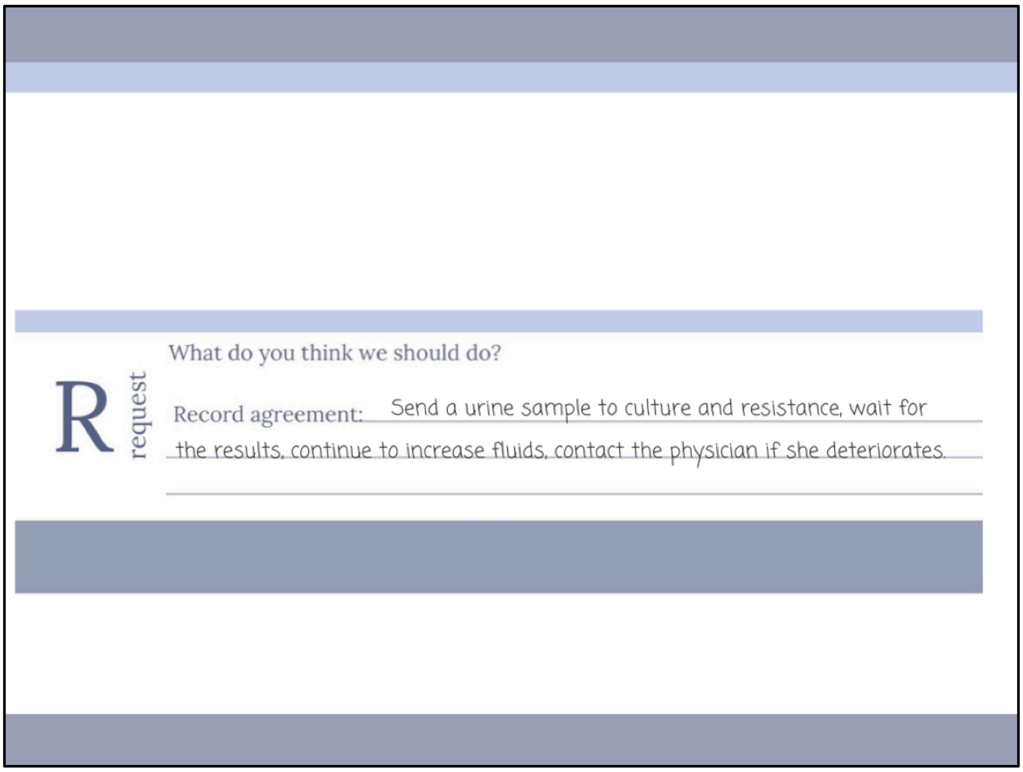

Next I ask the physician what to do.

Finally, there is room for your notes, if you need it.

Is there something that you would have done differently in the communication tool?

>Time for short discussion<

Then we will move on to Case 2 and now it is your turn to use the dialogue tool.

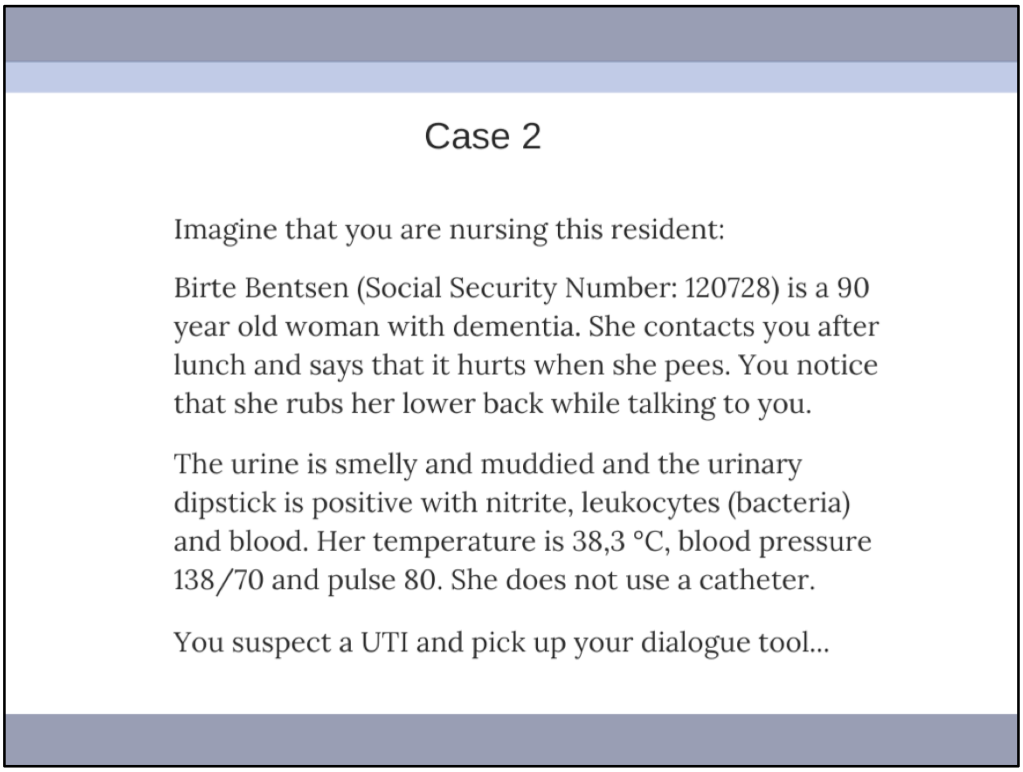

Here is the case:

>Read the case out loud<

Now you have to use the dialogue tool in front of you the same way I just did. We begin with page one for observations and reflection. In the third part, please discuss the four questions with your neighbour before we use the communication tool.

Try on your own or discuss with your neighbour if you are more comfortable with that. Please look up, when you are done, so I know when to move on.

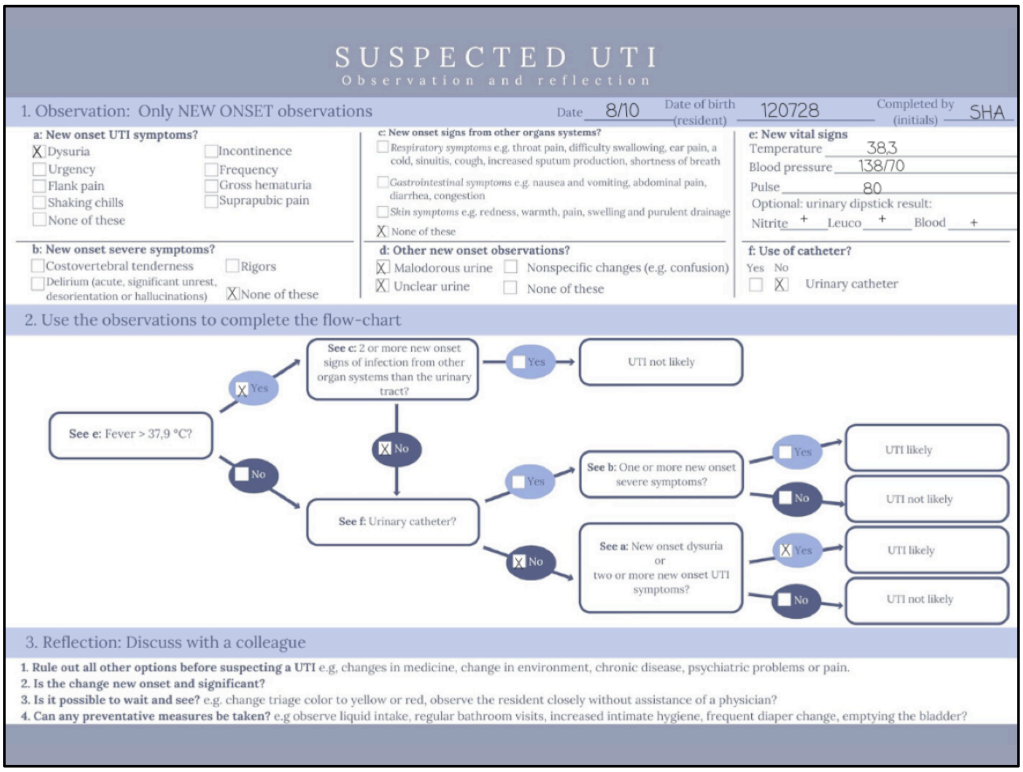

SUSPECTED UTI
Observation and reflection

1. Observation: Only NEW ONSET observations — Date 8/10 — Date of birth (resident) 120728 — Completed by (initials) SHA

a: New onset UTI symptoms?
- [X] Dysuria
- [ ] Urgency
- [ ] Flank pain
- [ ] Shaking chills
- [ ] None of these
- [ ] Incontinence
- [ ] Frequency
- [ ] Gross hematuria
- [ ] Suprapubic pain

c: New onset signs from other organs systems?
- [ ] Respiratory symptoms e.g. throat pain, difficulty swallowing, ear pain, a cold, sinuitis, cough, increased sputum production, shortness of breath
- [ ] Gastrointestinal symptoms e.g. nausea and vomiting, abdominal pain, diarrhea, congestion
- [ ] Skin symptoms e.g. redness, warmth, pain, swelling and purulent drainage
- [X] None of these

e: New vital signs
Temperature 38,3
Blood pressure 138/70
Pulse 80
Optional: urinary dipstick result:
Nitrite + Leuco + Blood +

b: New onset severe symptoms?
- [ ] Costovertebral tenderness
- [ ] Delirium (acute, significant unrest, desorientation or hallucinations)
- [ ] Rigors
- [X] None of these

d: Other new onset observations?
- [X] Malodorous urine
- [X] Unclear urine
- [ ] Nonspecific changes (e.g. confusion)
- [ ] None of these

f: Use of catheter?
Yes [ ] No [X] Urinary catheter

2. Use the observations to complete the flow-chart

See e: Fever > 37,9 °C? — [X] Yes / [ ] No
See c: 2 or more new onset signs of infection from other organ systems than the urinary tract? — [ ] Yes → UTI not likely / [X] No
See f: Urinary catheter? — [ ] Yes / [X] No
See b: One or more new onset severe symptoms? — [ ] Yes → UTI likely / [ ] No → UTI not likely
See a: New onset dysuria or two or more new onset UTI symptoms? — [X] Yes → UTI likely / [ ] No → UTI not likely

3. Reflection: Discuss with a colleague

1. **Rule out all other options before suspecting a UTI** e.g. changes in medicine, change in environment, chronic disease, psychiatric problems or pain.
2. **Is the change new onset and significant?**
3. **Is it possible to wait and see?** e.g. change triage color to yellow or red, observe the resident closely without assistance of a physician?
4. **Can any preventative measures be taken?** e.g observe liquid intake, regular bathroom visits, increased intimate hygiene, frequent diaper change, emptying the bladder?

This is how I have chosen to fill out page 1. In part 3 I would consider contacting the physician, but also if any preventive measures could be taken, so that the situation would not repeat itself.

Are there other considerations?

>Leave time in for discussion<

Let us continue with the communication tool. I know that the healthcare helpers are not actually going to contact the physician, but filling in page 2 will give you knowledge about what kind of information your colleagues will need. So please try to fill it anyway.

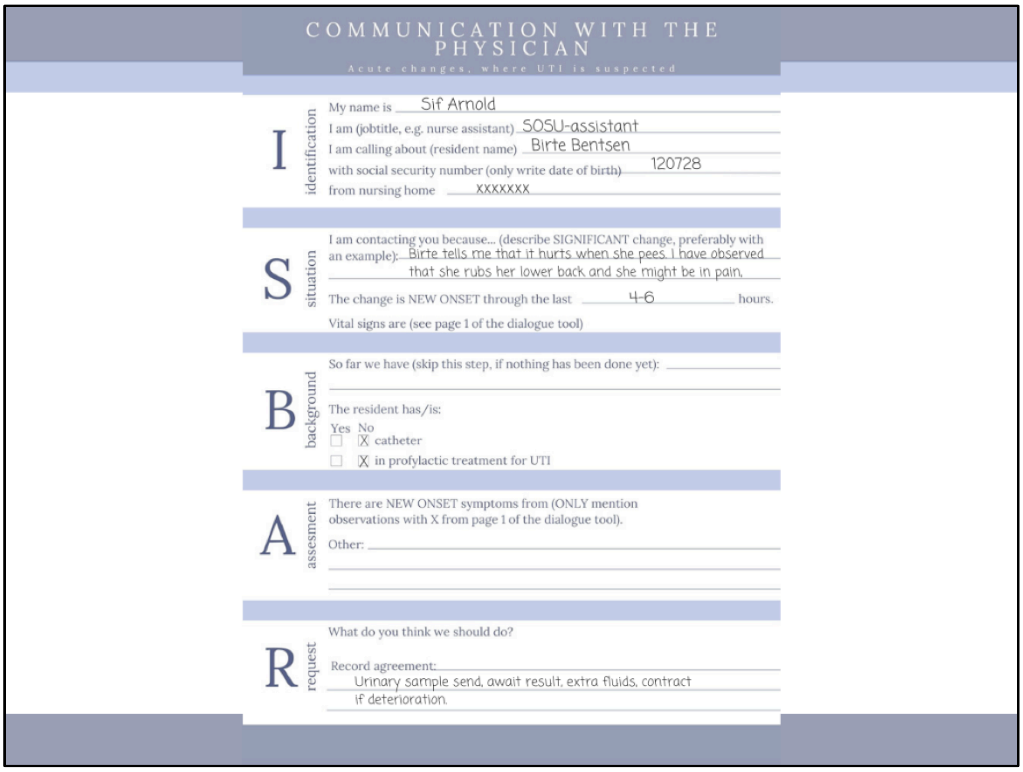

COMMUNICATION WITH THE PHYSICIAN

Acute changes, where UTI is suspected

I identification

My name is Sif Arnold

I am (jobtitle, e.g. nurse assistant) SOSU-assistant

I am calling about (resident name) Birte Bentsen

with social security number (only write date of birth) 120728

from nursing home XXXXXXX

S situation

I am contacting you because... (describe SIGNIFICANT change, preferably with an example): Birte tells me that it hurts when she pees. I have observed that she rubs her lower back and she might be in pain.

The change is NEW ONSET through the last 4-6 hours.

Vital signs are (see page 1 of the dialogue tool)

B background

So far we have (skip this step, if nothing has been done yet):

The resident has/is:

Yes	No	
☐	X	catheter
☐	X	in profylactic treatment for UTI

A assesment

There are NEW ONSET symptoms from (ONLY mention observations with X from page 1 of the dialogue tool).

Other:

R request

What do you think we should do?

Record agreement: Urinary sample send, await result, extra fluids, contract if deterioration.

This is how I would fill in page 2.

Has anybody done something other than I have?

>Time for short discussion<

Finally, I want to remind you when you need to use the dialogue tool and where the forms must go, when you have used them.

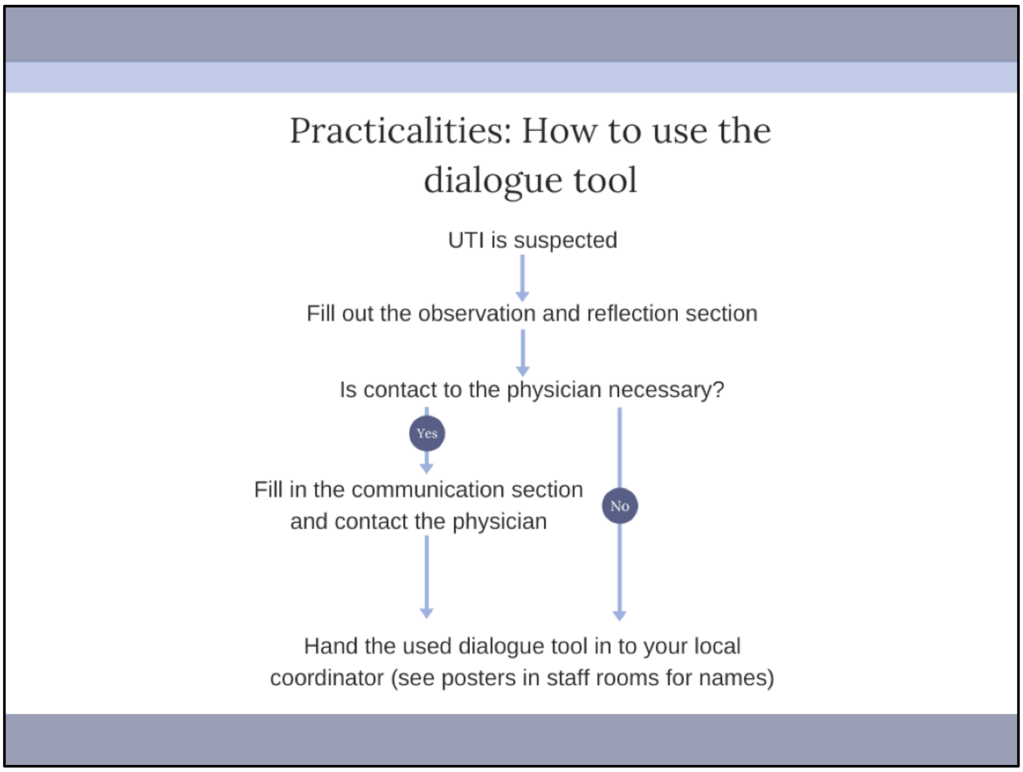

If you are in doubt or do not feel like you can complete the dialogue tool on you own, then ask a colleague for help. You can also take it in steps: It does not have to be the same person that starts the dialogue tool who finish it.

If you wait and see and the resident gets worse, then use the dialogue tool to update you observations to the present day.

Do you have any questions?

>Leave time for questions<

Thank you for your attention
