# Supplementary figures and images for: Development of a Tailored, Complex Intervention for Clinical Reflection and Communication about Suspected Urinary Tract Infections in Nursing Home Residents
Source: Antibiotics (Basel). 2020 Jun 25;9(6):360. doi: 10.3390/antibiotics9060360 (PMC7345997; doi:10.3390/antibiotics9060360)

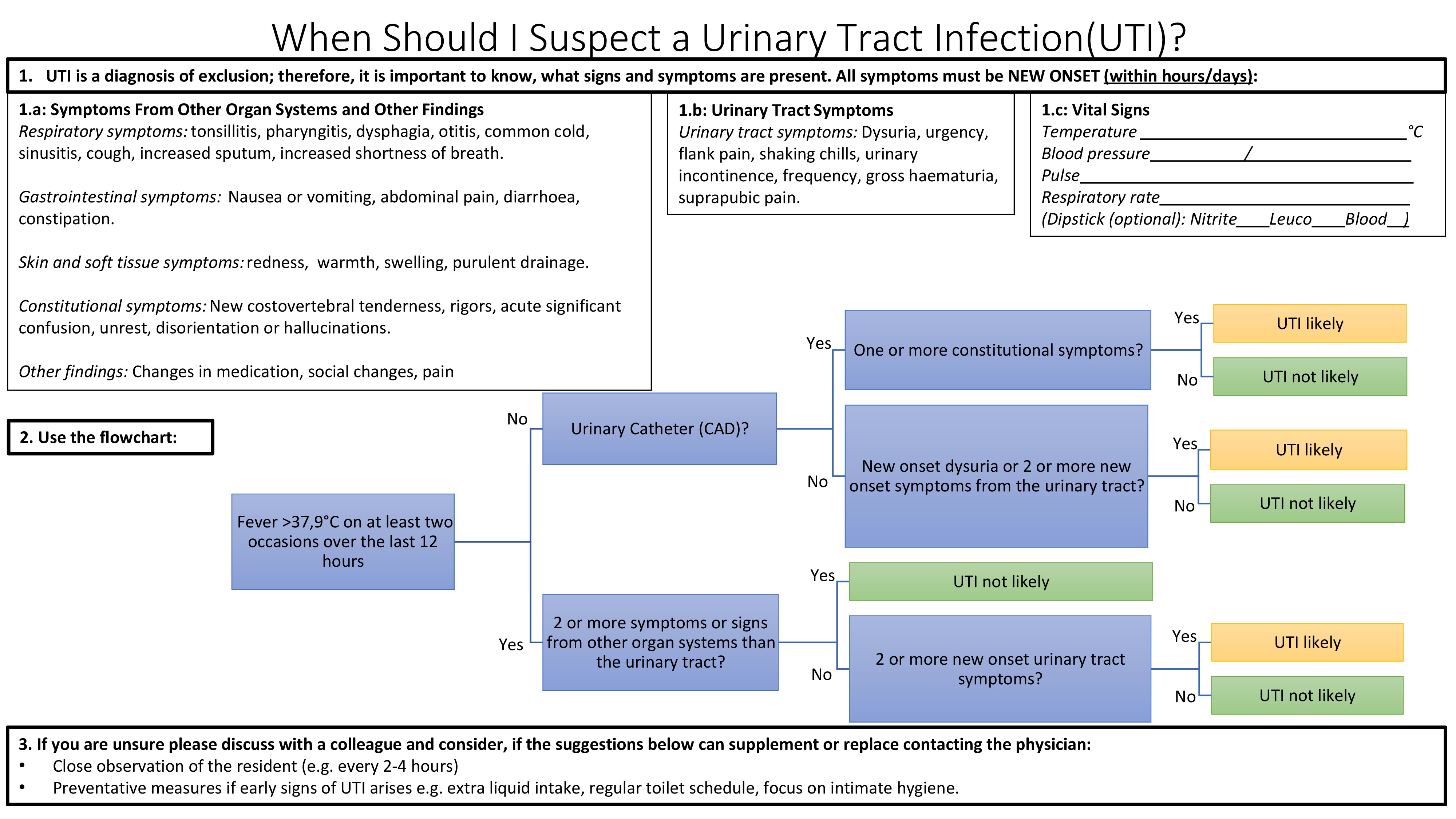

Supplement: Supplementary file 1 [file antibiotics-09-00360-s001.zip › supplementary materials/Figure S1_the first version of the reflection tool_phase 1 of the tailoring process.jpg]

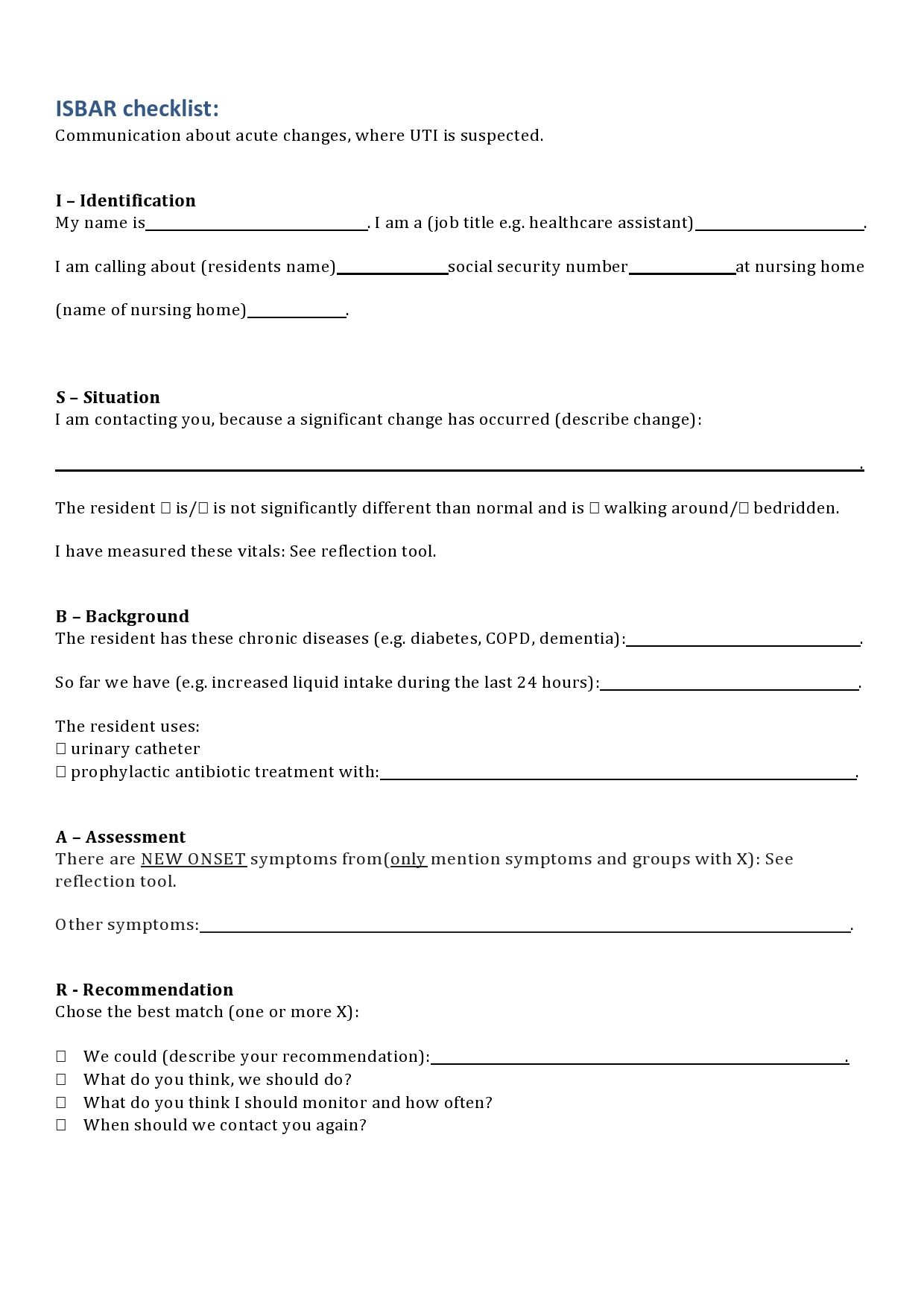

Supplement: Supplementary file 1 [file antibiotics-09-00360-s001.zip › supplementary materials/Figure S2_the first version of the communication tool_phase 1 of the tailoring process.jpg]

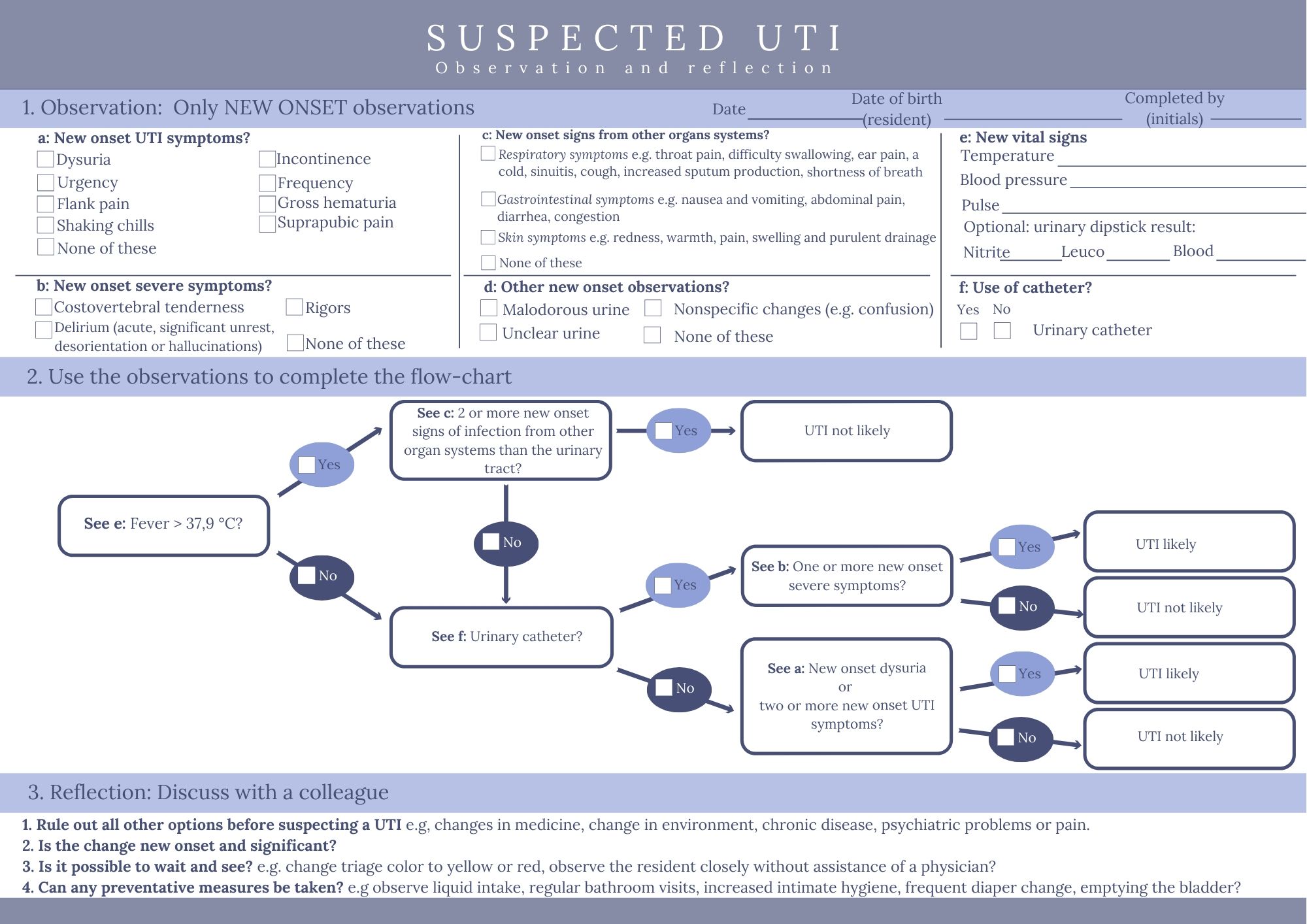

Supplement: Supplementary file 1 [file antibiotics-09-00360-s001.zip › supplementary materials/Figure S3_The Reflection Tool_Final.jpg]

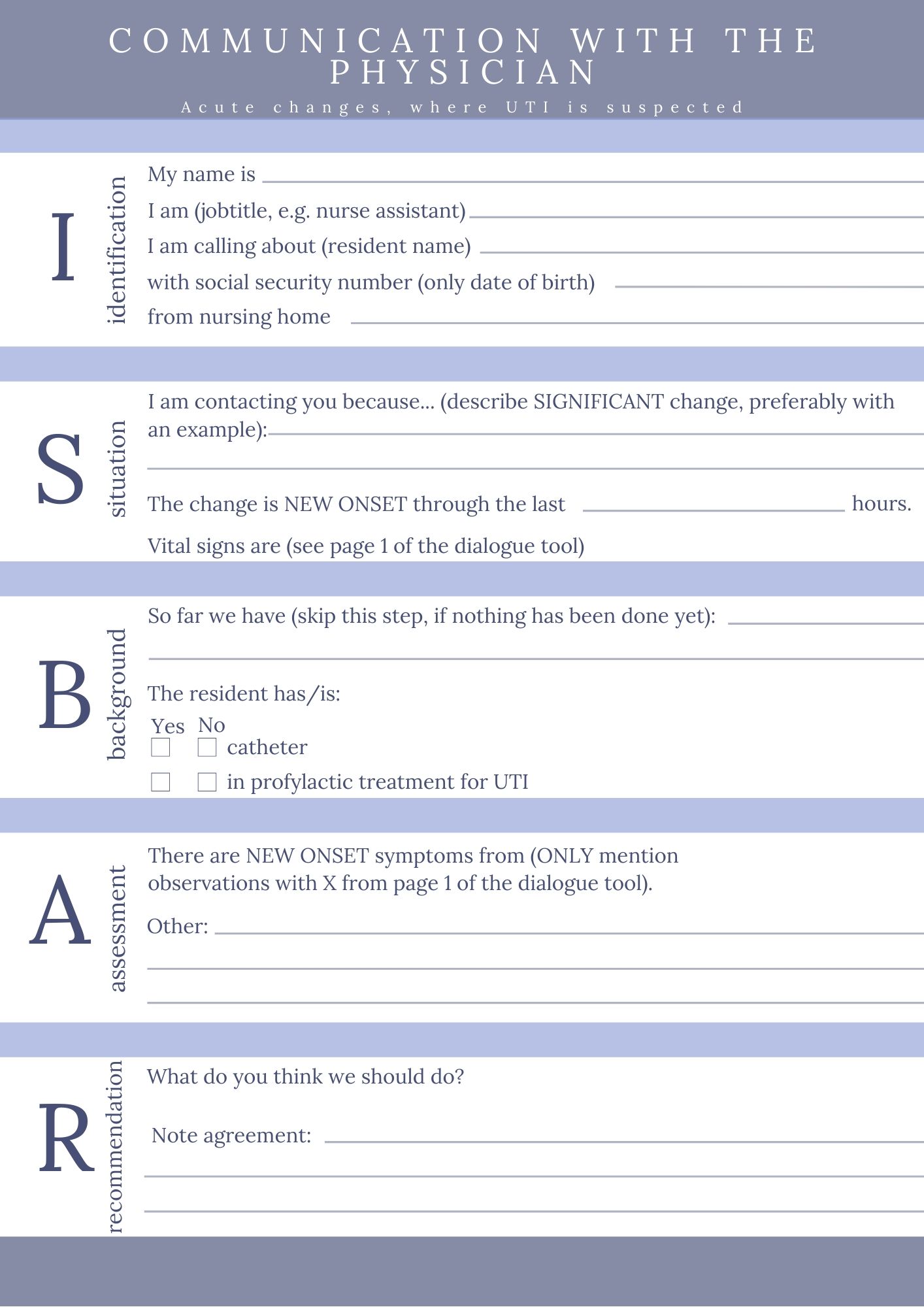

Supplement: Supplementary file 1 [file antibiotics-09-00360-s001.zip › supplementary materials/Figure S4_The communication Tool_Final.jpg]
